# Editorial: Active commuting: a strategy for improving student health in educational settings

**DOI:** 10.3389/fspor.2026.1844876

**Published:** 2026-04-20

**Authors:** Franscisco Javier Huertas Delgado, Ximena Palma-Leal, Rizka Maulida, Pablo Campos-Garzón

**Affiliations:** 1 Teacher Training Centre La Inmaculada, Universidad de Granada, Granada, Spain; 2 iGEO Group, School of Physical Education, Pontificia Universidad Católica de Valparaíso, Valparaíso, Chile; 3 Department of Epidemiology, Faculty of Public Health, Universitas Indonesia, Depok, Indonesia; 4 SPORT Research Group (CTS-2014), CIBIS (Centro de Investigación Para ElBienestar y la Inclusión Social) Research Center, University of Almería, Almería, Spain; 5 Department of Global Public Health, Karolinska Institutet, Stockholm, Sweden

**Keywords:** active transport, behavioral determinants, educational settings, movement behaviors, student health

## 1 Introduction

Educational settings represent key environments for promoting healthy behaviors among children, adolescents, and young adults (1, 2). Evidence suggests that physical activity starts to decline from childhood and continues to decline into adulthood (3, 4).

Among the healthy behaviors related to active lifestyles, active commuting to school or campus, mainly walking or cycling, has gained increasing attention as a promising and scalable public health approach (5, 6). By integrating physical activity into daily routines, active commuting allows individuals to increase their daily movement without requiring additional leisure time (7), while simultaneously contributing to broader societal goals such as environmental sustainability, reduced traffic congestion, and improved urban livability (8). Despite these potential benefits, active commuting behaviors vary widely across populations and academic contexts (9, 10). Understanding these factors is essential for designing effective interventions and policies aimed at promoting active lifestyles in educational settings.

The present Research Topic, Active Commuting: A Strategy for Improving Student Health in Educational Settings, brings together studies examining active commuting and related behavioral determinants among students across different educational stages and cultural contexts.

## 2 Overview of contributions

### 2.1 Active commuting and movement behaviors in educational settings

Martín-Moraleda et al. investigated the relationship between active commuting to school and adherence to the 24-Hour Movement Guidelines among Spanish adolescents. Their findings showed that more than half of the adolescents in the sample reported actively commuting to school, and those meeting a greater number of movement guidelines were more likely to commute actively. Notably, the association was particularly pronounced among girls. These results suggest that active commuting may serve as a meaningful entry point for improving integrated movement behaviors, including physical activity, sedentary behavior, and sleep, during adolescence.

Expanding the focus to the post-secondary context, Derkach et al. examined the prevalence and correlates of active commuting to campus among Canadian university and college students. Despite growing awareness of the benefits of active commuting, the prevalence of active commuting remained relatively modest in this large national sample. The study identified important correlates including age, gender, commute time, geographic context, and reported that active commuters were more likely to report meeting the Canadian physical activity guidelines. These findings highlight that active commuting patterns among university students are shaped by a complex interplay of personal characteristics, environmental factors, and institutional contexts. Furthermore, the transition to higher education is often associated with a decline in overall active behaviors, such as commuting modes that involve energy expenditure.

Understanding the motivations underlying active commuting behaviors is also crucial for designing effective interventions. Schönbach et al. addressed this need by validating the German Behavioral Regulation in Cycling to and from School Scale. Their study supported a three-factor structure with strong internal consistency and invariance across genders. Importantly, the motivational constructs captured by the scale were meaningfully associated with cycling behavior. By providing a reliable and validated measurement instrument, this work contributes valuable methodological tools for future research and interventions aimed at promoting cycling to school.

### 2.2 Psychological and behavioral determinants of student physical activity

While the previous contributions focus directly on commuting behaviors, other studies in this Research Topic provide complementary insights into the psychological and behavioral processes that influence students' engagement in physical activity more broadly.

Using machine learning approaches and a large sample of Chinese university students, Zhang et al. explored predictors of physical activity participation. Their findings identified exercise adherence, gender, sports skill mastery, and exercise motivation as key predictors of physical activity among students. Although the study did not specifically examine commuting behaviors, the results offer important insights into the broader determinants of active lifestyles among university students. In particular, the emphasis on motivation, competence, and behavioral persistence suggests that psychological and experiential factors play a central role in shaping students' engagement in movement-related behaviors. Complementing these findings, Tang et al. examined behavioral patterns in digital physical education among Chinese university students through the lens of the Theory of Planned Behavior. The study demonstrated that attitudes, subjective norms, and perceived behavioral control significantly predicted students' intentions to engage in digital physical education, with motivation partially mediating the relationship between intention and actual behavior. These studies reinforce the idea that promoting physical activity and active commuting in educational settings requires a multidimensional perspective. Behavioral choices are shaped not only by physical environments and commuting distances, but also by motivation, social influences, skills, and perceived opportunities.

## 3 Future directions and conclusions

Several important directions for future research emerge from this Research Topic. First, more longitudinal and intervention-based studies are needed to better understand causal pathways and evaluate strategies aimed at increasing active commuting among students. Within this context, university students emerge as a demographic with considerable potential not only for improving active commuting rates, but also for establishing lifelong active commuting behaviors into adulthood. Second, continued efforts to improve measurement tools, such as validated motivational scales and objective assessments of movement behaviors, will strengthen the evidence base and allow for more precise evaluation of interventions. Third, successful strategies to promote active commuting should integrate individual, social, and environmental components. Infrastructure improvements, school policies, and urban design interventions should be complemented by initiatives that enhance motivation, perceived competence, and social support for active commuting. Finally, research should continue to explore how active commuting interacts with broader daily movement behaviors, recognizing that students’ health is shaped by the combined balance of physical activity, sedentary behavior, and sleep.

In conclusion, the contributions to this Research Topic demonstrate that active commuting represents a promising, yet underutilized, opportunity to promote healthier lifestyles in educational settings. By integrating movement into daily routines, active commuting can support both individual health outcomes and broader societal goals. Advancing this field will require interdisciplinary approaches that combine behavioral science, education, urban planning, and public health to create environments where active commuting becomes a feasible and attractive choice for students across the educational lifespan.
